# Serological and Molecular Findings of *Leishmania* Infection in Healthy Donkeys (*Equus asinus*) from a Canine Leishmaniosis Endemic Focus in Tuscany, Italy: A Preliminary Report

**DOI:** 10.3390/pathogens8030099

**Published:** 2019-07-09

**Authors:** Simona Nardoni, Iolanda Altomonte, Federica Salari, Mina Martini, Francesca Mancianti

**Affiliations:** Dipartimento di Scienze Veterinarie, Università degli Studi di Pisa, 56124 Pisa PI, Italy

**Keywords:** donkey, *Leishmania*, seroprevalence, Italy, IFAT, PCR, emerging protozoa, new animal hosts

## Abstract

*Leishmania* parasites are considered to be emergent zoonotic pathogens, which is a new concept regarding their epidemiology and the identification of novel animal hosts. The present study is the first in Italy to evaluate anti *Leishmania* seroprevalence, and the first in Europe to detect parasite DNA in donkeys’ blood. The study was performed on jennies living in a *Leishmania infantum* endemic area of Central Italy. One hundred and ten blood samples were obtained from 67 healthy lactating Amiatina jennies that were semi-extensively reared in Tuscany. When possible, more than one sample was subsequently obtained from the same subject. All samples were processed by immunofluorescence antibody test (IFAT) and by polymerase chain reaction (PCR). For the results, 11 out of 30 animals (36.7%) showed positive scores under IFAT. In addition, 22 out of the other 37 jennies had positive scores, also. The animals showed titers ranging from 40 to 320. Furthermore, 2 subjects that were submitted for 2 and 3 blood samplings, both had more than one positive score. Moreover, 2 seropositive animals were positive for *Leishmania* DNA. Donkeys are considered to be a preferred source for a sandfly blood meal, even if clinical leishmaniosis has never been reported in Europe for this animal species. In the view of these facts, our preliminary findings would suggest the role of donkey as a potential reservoir for this protozoan agent. Additional studies would be welcome to elucidate the role of the donkey in *Leishmania* epidemiology of CanL endemic areas and to confirm the preliminary findings and the hypothesis proposed here.

## 1. Introduction

*Leishmania infantum* is a protozoan parasite responsible for canine leishmaniosis (CanL) and infantile kala-azar in Italy and in Mediterranean countries [1], with dogs as the main reservoirs for the parasite. In South European endemic areas, other infected carnivores have been reported, in particular cats [2,3,4], foxes [5,6,7], wolves [8], mustelids, and viverrids [9]. 

On the other hand, rodents such as black rats [10] and hares [11,12,13] have previously been described as being infected. Moreover, bovine [14] and horses were proven to be affected by cutaneous leishmaniosis in both Central [15,16] and Southern Europe [17,18,19]. Wallabies and orangutans have also recently been recognized as being affected, in Spain [20,21].

Donkeys are reported to develop clinical leishmaniosis [22,23,24] in South America; *Leishmania braziliensis* was recognized as aetiological agent [25] and equids such horses, donkeys and mules are suspected reservoir for *L. braziliensis* in endemic areas [26]. Furthermore, donkeys were shown to be sensitive to infection from *Leishmania chagasi* (*L. infantum*), showing positive serology and occurrence of parasites in tissues, although with a negative xenodiagnosis [27]. Recent studies carried out in *Leishmania donovani* endemic foci in Ethiopia detected a high seropositivity rate in donkeys [28], parasite DNA and a widespread exposure to sandfly saliva [29]. Just recently, in Portugal, 1 donkey out of 186 was found to be seropositive [30]. Furthermore, *Phlebotomus orientalis*, a proven vector of *L. donovani,* showed zoophilic feeding preferences, with a predominant choice for bovine, followed by donkey [31], indicating that this latter species is a preferred source for bloodmeal, with a higher engorgement rate for the sandfly female [32]. 

Tuscany (Central Italy) is an endemic area for canine leishmaniosis. In this region, donkey breeding is extensively managed, and autochthonous breeds, which are employed in recreational activities, onotherapy and milk production, are preserved.

Data on the occurrence of *Leishmania* infection in donkeys from Europe are very rare. For these reasons, the aim of the present study was to evaluate the presence of parasite DNA in buffy coat and of specific antibodies in serum from jennies, living in a CanL endemic area in Tuscany.

## 2. Results

The results showed that 33 specimens out of 110 scored IFAT positive, with titers ranging from 40 to 320. In detail, 11 animals out of 30 (36.7%) showed an antibody response. In addition, 22two out of the other 37 jennies also obtained positive scores. Moreover, 2 animals that were submitted to 2 and 3 blood samplings, respectively, had positive results for both samplings. 

Blood specimens collected in June scored positive for *Leishmania* DNA in two animals, with weak IFAT titers.

Serological and molecular findings, along with sampling time, are reported in Table 1.

## 3. Discussion

The seroprevalence of anti-*Leishmania* antibodies in the present study was strikingly high (36.7%), in a population of healthy jennies. All of the subjects submitted to repeated sampling showed fleeting antibody titers. Most of them in fact, scored positive once, except for 2 animals that developed a further response after an apparent winter negativization, when examined during the transmission season. This finding would suggest a possible parasite clearance, which might be due to an effective immunological response, corroborating the hypothesis of an intrinsic species resistance of donkeys to *Leishmania* parasites. IFAT was selected for serological evaluation, following similar studies performed on horses from Brazil [33], even if its accuracy in the diagnosis of canine leishmaniasis is reported as declining in samples from endemic regions [34]. 

Our findings would allow us to hypothesize that *P. perniciosus*, the mostly occurring sandfly species in the study area [35,36], may have a host-feeding preference for donkeys, according to Gebresilassie et al. [31].

To the best of our knowledge, the present study is the first in Europe to evaluate the presence of *Leishmania* DNA in donkeys’ blood, and the first in Italy referring to serological data in this animal species, with a transversal study from Portugal being the only previous European record, with a seroprevalence of about 0.5%. [28] In the present study, striking seroprevalences in this animal species, with a longitudinal evaluation of serological changes, were reported. 

In a similar study carried out on healthy horses from Spain [37] a lower seroprevalence was reported, moreover, specific lymphocyte proliferation was observed in 36.4% of the investigated animals, suggesting, along with the low occurrence of clinical patent leishmaniosis, the efficacy of the cell mediated immune response. 

The high seroprevalence, together with the lack of reports of *L. infantum* clinical cases in donkey in Europe, would suggest a strong resistance of this animal species versus the parasite, as well as a high exposure degree to infected vectors. Buffy coat for *Leishmania* does not represent the most sensitive tissue, so the detection of 2 positive samples would indicate an active parasite circulation among the tested jennies. Our finding would suggest the role of this species as a potential reservoir for this protozoan agent.

## 4. Materials and Methods 

A total of 110 serum samples (1 mL each) were obtained from 67 healthy lactating Amiatina jennies, aged from 4 to 18 years. The animals were semi-extensively reared near Scarlino (42°54′28″N 10°51′05″E), Tuscany, in an area where canine leishmaniosis has a seroprevalence of about 20% (unpublished data). All the animals were living in the breeding farm for at least two transmission seasons. Repellent and insecticidal treatments were not applied to all of the donkeys. Dogs were neither present in the farm, nor in the wider environment.

Sera from 30 jennies, already serologically processed for *Toxoplasma gondii* and piroplasms, were used in the present study in order to estimate a seroprevalence rate. Blood specimens from the remaining animals were collected to carry out genetic analysis about dairy genes. The samples were obtained from 37 animals. If available, specimens were drawn at about 3, 6 and 10 months after parturition. In detail, 15 of 37 animals had a unique blood sampling, 1 jenny yielded 2 specimens, and from the other 21 animals, 3 blood samples were obtained. Furthermore, blood samples added to Ethylenediaminetetraacetic acid (EDTA) were drawn from the 37 animals for parasitological molecular testing.

All serum samples were used to carry out IFAT in order to detect anti-*Leishmania* antibodies. The testing was conducted as previously reported for canine leishmaniosis [38] diagnosis, using an anti-horse IgG FITC (Sigma Aldrich, Italy) as a secondary antibody, diluted 1:30. The cut-off value was put at 1/40, as reported by Soares et al. [39]. 

Whole blood samples were processed for molecular purposes. DNA was extracted from 200 µL of buffy coat by blood/cultured cells genomic DNA extraction mini kit (Trevose, PA, USA) and all samples were submitted to PCR assay. For detection of Leishmania DNA, the ITS1 gene was selected as PCR target, as previously described [13]. PCR amplifications were performed in 50 μL of reaction mixture, containing 200 μM of deoxynucleoside triphosphates, 0.5 μM of each primer, 1.25 U of Taq polymerase (Lucigen Corporation, Middleton, Wisconsin, USA), and 2 μL of genomic DNA. All amplifications were performed using an automated thermal cycler Gene-Amp PCR System 2700 (Perkin Elmer, Norwalk, Connecticut, USA).

PCR products were then analyzed by electrophoresis on 1.5% agarose gel at 100 V for 45 min; the gel was stained with ethidium bromide and observed. SharpMassTM 100 Plus Ladder (Euroclone, Milano, Italy) was used as a DNA marker.

## 5. Conclusions

The preliminary results obtained in the present paper show a high seroprevalence for *Leishmania* in examined donkeys, as well as the occurrence of parasite DNA in blood. Additional studies would be welcome, both to further investigate the kinetics of antibody response over a longer time and to elucidate the role of donkey in *Leishmania* epidemiology of CanL endemic areas, in order to confirm the preliminary findings and the hypothesis proposed here. 

## Figures and Tables

**Table 1:** Age, sampling time and serological findings of positive animals.

N. Jenny	Age (years)	Month of Sampling	IFAT Titer
1	15	July 2016	1/80
2	7	July 2016	1/80
3	5	July 2016	1/80
4	12	July 2016	1/40
5	17	July 2016	1/40
6	4	July 2016	1/40
7	9	July 2016	1/40
8	5	July 2016	1/80
9	10	July 2016	1/40
10	4	July 2016	1/80
11	10	July 2016	1/80
12	13	June 2017	1/160
**N. Jenny**	**Age (years)**	**Month of Sampling**	**IFAT Titer**
12		March 2018	neg
13	10	July 2017	1/80
14	10	June 2017	1/80
14		October 2017	neg
14		May 2018	neg
15	9	November 2017	1/80
15		March 2018	neg
16	17	January 2018	1/320
16		July 2018	neg
16		October 2018	neg
17	10	June 2017	1/160
18	6	October 2017	1/160
18		March 2018	neg
18		July 2018	1/40
19	6	June 2017	1/80*
19		October 2017	neg
19		March 2018	neg
20	5	January 2018	neg
20		June 2018	1/40
20		October 2018	1/160
21	5	May 2017	1/80
21		October 2017	1/80
21		March 2018	neg
22	5	November 2017	neg
22		June 2018	1/160
22		October 2018	neg
23	5	November 2017	neg
23		May 2018	neg
23		October 2018	1/80
24	5	October 2017	1/160
24		January 2018	1/320
24		June 2018	neg
25	4	July 2018	neg
25		May 2018	neg
26	4	June 2017	1/160
26		October 2017	neg
26		May 2018	neg
27	4	July 2017	neg
27		December 2017	neg
27		May 2018	1/160
28	4	October 2017	1/160
**N. Jenny**	**Age (years)**	**Month of Sampling**	**IFAT Titer**
28		January 2018	neg
28		June 2018	neg
29	13	June 2017	1/40 *

Legend: * PCR positive specimens.
